# Rapid detection of Mycobacterium tuberculosis based on cyp141 via real-time fluorescence loop-mediated isothermal amplification (cyp141-RealAmp)

**DOI:** 10.3389/fcimb.2024.1349063

**Published:** 2024-06-13

**Authors:** Yinyin Zhu, Zi Feng, Yinfang Xu, Sha Luo, Ruixian Zhang, Xudong Shi, Xuping Wu, Hongying Zhang

**Affiliations:** ^1^ Department of Microbial Testing, Nanjing Center for Disease Control and Prevention Affiliated to Nanjing Medical University, Nanjing, Jiangsu, China; ^2^ School of Public Health, Nanjing Medical University, Nanjing, Jiangsu, China; ^3^ Department of Infectious Diseases, the Affiliated Zhongda Hospital of Southeast University, Nanjing, Jiangsu, China; ^4^ The Second Hospital of Nanjing, Nanjing University of Chinese Medicine, Nanjing, Jiangsu, China

**Keywords:** tuberculosis, Mycobacterium tuberculosis, LAMP, Xpert MTB/RIF ; cyp141-RealAmp, Mycobacterium tuberculosis complex, *Mycobacterium bovis*, cytochrome P450

## Abstract

**Background:**

The rapid detection of Mycobacterium tuberculosis (MTB) is essential for controlling tuberculosis. **Methods** We designed a portable thermocycler-based real-time fluorescence loop-mediated isothermal amplification assay (cyp141-RealAmp) using six oligonucleotide primers derived from cyp141 to detect MTB. A combined number of 213 sputum samples (169 obtained from clinically diagnosed cases of pulmonary TB and 44 from a control group without tuberculosis) underwent Acid-fast bacillus (AFB) smear, culture, Xpert MTB/RIF assays, and cyp141-RealAmp assay.

**Results:**

By targeting MTB cyp141, this technique could detect as low as 10 copies/reaction within 30 min, and it was successfully rejected by other mycobacteria and other bacterial species tested. Of the 169 patients, there was no statistical difference between the detection rate of cyp141-RealAmp (92.90%, 95% CI: 89.03–96.07) and that of Xpert MTB/RIF (94.67%, 95% CI: 91.28–98.06) (*P* > 0.05), but both were statistically higher than that of culture (65.68%, 95% CI: 58.52–72.84) (*P*< 0.05) and AFB (57.40%, 95% CI: 49.94–64.86) (*P*< 0.05). Both cyp141-RealAmp and Xpert MTB/RIF had a specificity of 100%. Furthermore, a high concordance between cyp141-RealAmp and Xpert MTB/RIF was found (*Kappa* = 0.89).

**Conclusion:**

The cyp141-RealAmp assay was shown to be effective, responsive, and accurate in this study. This method offers a prospective strategy for the speedy and precise detection of MTB.

## 1 Introduction

In most developed nations, tuberculosis (TB) is nearly eradicated as a communicable illness. However, developing countries still encounter substantial challenges due to the significant burden and expenses associated with TB. Based on the Global Tuberculosis Report by the World Health Organization (WHO) in 2022, India holds the top position worldwide in terms of the quantity of tuberculosis patients, while China secures the third spot. The report from WHO reveals that the global death toll from TB reached 1.6 million individuals, encompassing 187,000 individuals who were HIV-positive (W.H.O, 2022). The HIV and COVID-19 epidemics have exacerbated the situation in nations grappling with a significant tuberculosis burden.

TB, primarily caused by Mycobacterium tuberculosis (MTB), has emerged as a major contributor to mortality worldwide. However, it is possible that *Mycobacterium bovis* (*M. bovis*), which belongs to the Mycobacterium tuberculosis complex (MTBC), could be implicated (Ansumana et al., 2017). In 2016, 147,000 cases of zoonotic tuberculosis caused primarily by *M. bovis* were reported globally (W.H.O, 2016; Taye et al., 2021). Due to the challenges in distinguishing *M. bovis* from MTB during diagnoses, the global prevalence of zoonotic tuberculosis is likely to be underestimated (Devi et al., 2021). When comparing TB caused by MTB infections to *M. bovis*, there are notable variations in the recommended antituberculosis drugs and treatment duration (Gallo et al., 2016). Therefore, MTB must be detected quickly and distinguished from other MTBC members in order to be diagnosed, treated, and controlled (Han et al., 2007).

However, current diagnostic methods are far from meeting the requirements. Clinicians commonly use Acid-fast bacillus (AFB) and culture-based routine diagnostics for MTB. The gold standard for diagnosing active TB is a positive MTB culture from clinical samples, but a diagnosis of MTB can take up to 6–8 weeks due to its slow growth (Suarez et al., 2019). AFB is fast, but it lacks sensitivity and is incapable of distinguishing tuberculosis from non-tuberculosis mycobacterial (NTM) infections (Acharya et al., 2020). The WHO has recommended the use of molecular nucleic acid amplification test (NAAT) for detecting MTB in TB cases, including line probe assays (LPA), multiplex PCR, and Xpert MTB/RIF (Maclean et al., 2020). Nevertheless, in areas with limited resources, these NAAT tests are not accessible due to their high cost and the need for specialized equipment and technical expertise.

A commercial iteration of loop-mediated isothermal amplification (TB-LAMP), was created by Eiken Chemical Co., Ltd, distinguishing it from other NAATs. Thermal cycling in these assays lacks precise control; instead, they are incubated at a consistent temperature. The strand displacement technique employs high-strand displacement DNA polymerase along with two specifically crafted inner and outer primers (Shete et al., 2019). The TB-LAMP product is recognized by visual inspection, which may lead to misinterpretation (Sethi et al., 2013). Real-time fluorescence loop-mediated isothermal amplification (RealAmp), a technology that amplifies nucleic acid sequences, is an advancement over LAMP technology. It utilizes a nucleic acid dye to produce a fluorescence signal that can be observed in real time (Ou et al., 2019), thereby minimizing human error. Using DNA intercalating dyes like SYBR Green I carries the potential for obtaining inaccurate positive outcomes due to their ability to attach to any double-stranded DNA molecule, regardless of its size (Das et al., 2022). Calcein is the most commonly used dye for the colorimetric detection of LAMP. At first, calcein binds with Mn^2+^ ions, reducing its fluorescence activity and causing the LAMP reaction mixture to turn orange. Later, Mn^2+^ ions form a complex with pyrophosphate P_2_O_7_^4-^, restoring green fluorescence during amplification. Additionally, calcein–Mg^2+^ complex formation further enhances the fluorescence signal (Das et al., 2022). So, the amplification reaction of calcium fluorescein may be used to produce fluorescent characteristics and use it as a real-time fluorescent indicator.

There have been a variety of targets described for the detection of MTB, such as genes encoding the 32-kDa proteins (Soini et al., 1994), mtp40 (Khosravi et al., 2017), and rpoB (Xia et al., 2022), as well as insertion sequence (IS) elements such as IS1081 or IS6110 (Nyaruaba et al., 2020). Among these targets, IS6110 is found exclusively in MTB, which is the most abundant and best characterized (Antoine et al., 2021). It has become an essential diagnostic tool for differentiating MTB from other mycobacteria (Sam et al., 2021; Shanmugakani et al., 2021). However, reports showed it is absent in some MTB strains and is also present in some other members of MTBC, which can result in false negative or false positive outcomes (Fomukong et al., 1994; Soto et al., 2012).

Cychrome P450 (Cyp) is a family of iron-containing hemoproteins. In MTB, the cytochrome P450 141 (cyp141) is an important virulence factor (Salehi et al., 2018). There are 16 regions of differences (RD) in the genomes of MTB and *M. bovis* or Bacillus Calmette Guerin (*BCG*), and the cyp141 gene with a base length of 1203-bp is located in one of the RD (Heidari et al., 2015). A study by Darban found that MTB can be directly detected from respiratory specimens by using the cyp141 gene (Darban-Sarokhalil et al., 2011). The cyp141 has been suggested for detecting MTB in clinical samples (Darban-Sarokhalil et al., 2013; Sadr et al., 2017). In a study, it was found that the cyp141 gene was more sensitive than the commonly used IS6110 gene. But both genes have an equal specificity of 100% (Farzam et al., 2015). However, conflicting results have emerged regarding the specificity of detecting different strains of bacteria. According to Darban’s report, the cyp141 gene was found to be partially present in both *M. bovis* and *BCG* (Darban-Sarokhalil et al., 2011), whereas Farzam et al. utilized the mentioned primers.did not bind to *M. bovis* (Farzam et al., 2015). These studies suggest that researchers need to design specific primers based on differential nucleic acid segments to distinguish between MTB-infected individuals, *M. bovis*-infected individuals, or *BCG*-vaccinated individuals.

A RealAmp was designed for the rapid and efficient identification of MTB by utilizing custom oligonucleotide primers targeting distinct segments of cyp141. Therefore, this study aims to develop a more reliable RealAmp assay based on a portable thermocycler, termed cyp141-RealAmp, to provide a more reliable diagnostic tool for the identification of clinical tuberculosis.

## 2 Materials and methods

### 2.1 Preparation of standardized plasmids

Sangon Biotech Co., Ltd (Shanghai, CN) synthesized a 1203 bp gene fragment for cyp141 (Gene ID: 887409). Using the formula below, it was calculated that the plasmid would have the following copy number:


$$\begin{array}{l} \text{Amount }\left(\text{copies}/\text{µl}\right)\;=\frac{\text{Plasmid concentration }\left(\text{ng}/\text{µl}\right)\;\times \;10^{-9}\;\times \;6.02\;\times \;10^{23}}{\text{DNA length in base paris }\times \;660}\\ \;=\frac{93\times \;10^{-9}\;\times \;6.02\;\times \;10^{23}}{3889\;\times \;660}=2\times 10^{9}\left(\text{copies/μl}\right) \end{array}$$

### 2.2 Design of primers for cyp141-RealAmp

**Figure 1:**
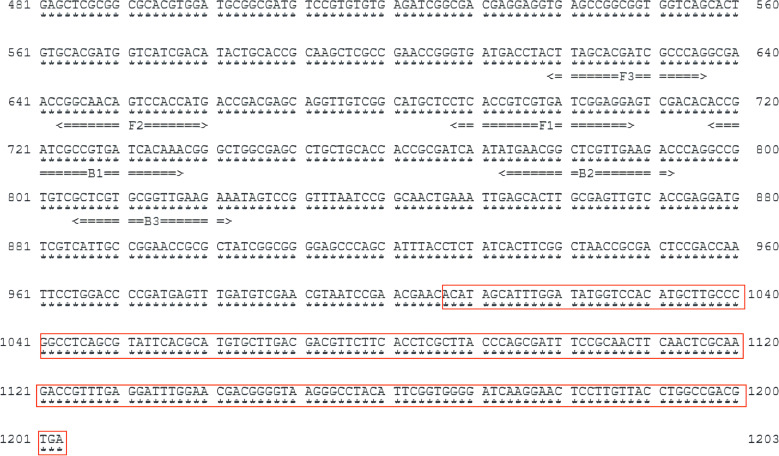
Location and sequence of the selected gene (part of cyp141) used to design amplification primers. The red box shows the overlapping parts of the cyp141 gene in the H37Rv and *M. bovis* or *BCG* strains.

The sequence for the gene cyp141 (Rv3121) was acquired from the NCBI database (https://www.ncbi.nlm.nih.gov/). According to LAMP guidelines of “Primer Explorer” (http://primerexplorer.jp/e/), the software provides several sets of primers for cyp141, multiple comparisons were performed in NCBI to design the primers to ensure their specificity for MTB. After conducting initial research, a collection of extremely responsive primers was chosen, and loop primers were subsequently designed for the selected primer. **Table 1** and **Figure 1** display the information regarding primer design, premier sequences, and the positions of cyp141-RealAmp primers. Sangon Biotech Co., Ltd (Shanghai, CN) provided the oligonucleotide primers.

**Table 1:** Primers used in the present study.

Primer Name	Sequence (5’-3’)	Length
FIP	TCCTCCGATCACGACGGTGAGCGGCAACAGTCCACCATG	39
BIP	ACCGATCGCCGTGATCACAAAGTCTTCAACGAGCCGTTCAT	41
LF	GCATGCCGACAACCTGC	17
LB	TGCACCACCGCGATCAA	17
F3	CTTAGCACGATCGCCCAG	18
B3	TTCTTCAACCGCACGAGC	18

### 2.3 Cyp141-RealAmp assay

The cyp141-RealAmp reaction (25 µl) was prepared by using 1.6 µM of each inner primer, 0.2 µM of each outer primer, and 0.8 µM of each loop primer. Additionally, 2.5 µl of 10 × Isothermo buffer (Mg^2+^-free) from Bio Lebo Biotechnology in Beijing, CN, 1.4 mM dNTPs from GeneRay Biotechnology in Shanghai, CN, 8 mM Mg^2+^ from Bio Lebo Biotechnology in Beijing, CN, 8 U of Bst DNA polymerase 3.0 from Bio Lebo Biotechnology in Beijing, CN, 1 µl of fluorescent indicators (Calcein-Mn^2+^), and 5 µl of DNA were included in the reaction mixture. Positive control was MTB H37Rv DNA, while negative control was RNase-free water. The reaction program was 65°C for 30 min and using the thermostatic fluorescence detector Genie II (OptiGene, UK).

### 2.4 Analytical specificity and limit of detection of the assay

The optimized primers were used for subsequent assays. Genome DNA from all cultures was extracted using the DNA kit of bacteria (Omega, USA) as instructed by the manufacturer. To ensure future usability, the DNA samples were preserved at a temperature of -20°C. The specificity of cyp141-RealAmp was detected by using DNA extracted from MTB H37Rv, *BCG*, *M. bovis*, and 10 NTM strains (*M. avium*, *M. landraceum*, *M. schlegelii*, *M. kansasii*, *M. asiaticum*, *M. scrofula*, *M. gordonii*, *M. incidentalis*, *M. grasseri* and *M. intracellulare*), and 5 non-mycobacterial species (*Nocardia brasiliensis*, *Corynebacterium beijingense*, *Pneumococcus pneumoniae*, *Legionella pneumophila*, *Bordetella pertussi*). The Chinese Center for Disease Control and Prevention (Beijing, CN), Chengdu Institute of Biological Products Co., Ltd (Chengdu, CN), The Second Hospital of Nanjing (situated in Nanjing, CN), and National Institutes for Food and Drug (Beijing, CN), supplied those strains of bacteria. More details are listed in **Table 2**.

**Table 2:** Strains used for specificity testing.

Strain	No. of strains	cyp141-RealAmp	Strain origin
**MTB H37Rv**	1	+	Chinese Center for Disease Control and Prevention
***M. bovis***	4	–	The Second Hospital of Nanjing
***BCG***	1	–	Chengdu Institute of Biological Products Co., Ltd
***M. intracellulare***	1	–	The Second Hospital of Nanjing
***M. avium***	1	–	National Institutes for Food and Drug
***M. landraceum***	1	–	
***M. schlegelii***	1	–	
***M. kansasii***	1	–	
***M. asiaticum***	1	–	
***M. scrofula***	1	–	
***M. gordonii***	1	–	
***M. incidentalis***	1	–	
***M. grasseri***	1	–	
***Nocardia brasiliensis***	1	–	
***Corynebacterium beijingense***	1	–	
***Pneumococcus pneumoniae***	1	–	
***Legionella pneumophila***	1	–	
***Bordetella pertussis***	1	–	

The ability of the cyp141-RealAmp assay to detect low levels was evaluated by testing the diluted standard plasmid of cyp141 gene (ranging from 2×10^4^∽2×10^0^ copies/μl and 1 copy/μl, with 10^5^, 10^4^, 10^3^, 10^2^, 10^1^, and 5 copies per reaction) in the cyp141-RealAmp reaction system. RNase-free water was utilized as a negative control (NC) in place of the template.

### 2.5 Investigation of clinical performance

Sputum samples from 213 suspected pulmonary TB cases from August 2022 to December 2022 were collected from the Nanjing Second Hospital Laboratory Center. The health industry-standard Diagnosis for pulmonary tuberculosis (WS 288‐2017) (National Health and Family Planning Commission of the People’s Republic of China, 2018) serves as the foundation for all diagnostics; and the basic clinical information of patients is shown in the **Supplementary Table S1**. The collected sputum samples were divided into four parts for processing. One portion was subjected to AFB staining to detect TB bacilli, while another portion was cultured on Lowenstein-Jensen medium at 37°C for six to eight weeks, following the manufacturer’s instructions (Baso, China). Culture-positive samples were identified using standard microbiological techniques (Giampaglia et al., 2005). Moreover, a third portion of the sputum samples was tested using Xpert MTB/RIF (Cepheid, USA).

The final portion of each sputum samples was mixed with a 1–2 times volume of 4% NaOH solution, adjusted based on the sample’s volume and characteristics, then shaken and mixed, and allowed to sit at room temperature for 15 min until the sample liquefied and became slightly clear. Approximately 2–3 ml of the liquid sample were placed into the tube for centrifugation at a speed of 13,000 revolutions per minute for a duration of 10 min. The supernatant was discarded, and then 10 µl of proteinase K was introduced into each tube and maintained at a temperature of 100°C for 10 min. Finally, the DNA was purified according to the MicroElute™ DNA Clean-up system instructions (Omega, USA). DNA extracted from the sputum was stored at -20°C. All manipulations were taking place in a biosafety cabinet.

In the paper, all the parts involving personal privacy (such as name, identification number, address, and so on) are de-labeled, and the specific information of subject is not reported in the paper. Approval for this study was granted by the ethics committee at Nanjing Center for Disease Control and Prevention, with reference number PJ2020-A001–04.

### 2.6 Analyses statistical

Despite the gold standard of MTB culture isolation, none of the laboratory diagnosis methods are 100% accurate. Therefore, confirmed TB cases were utilized as the reference standard for calculating various parameters. The *Kappa* statistic determined the concordance between cyp141-RealAmp and Xpert MTB/RIF. SPSS 26.0 was used to analyze the data.

## 3 Results

### 3.1 Specificity of cyp141-RealAmp assay

**Figure 2:**
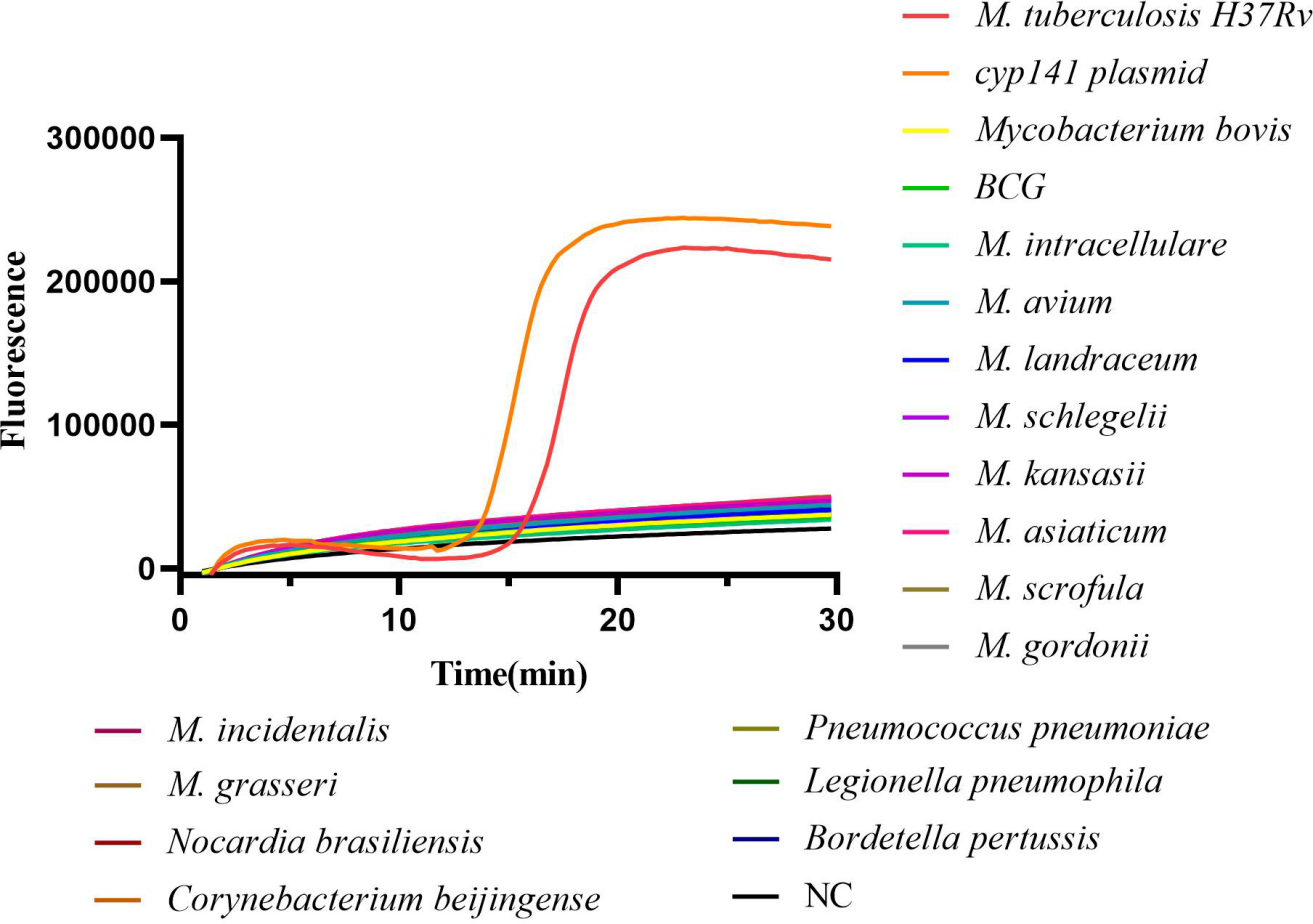
Analytical specificity of the cyp141-RealAmp for Mycobacterium tuberculosis.

The specificity of the cyp141-RealAmp was confirmed using template DNA isolated from MTB H37Rv, *M. bovis*, *BCG*, 10 NTM strains, and 5 non-mycobacterial species. Positive amplification was only observed for MTB H37Rv and cyp141 standard plasmid in 30 min, not for *M. bovis*, *BCG*, NTM strains and non-mycobacterial species, as shown in **Figure 2**.

### 3.2 Limit of cyp141-RealAmp assay

**Figure 3:**
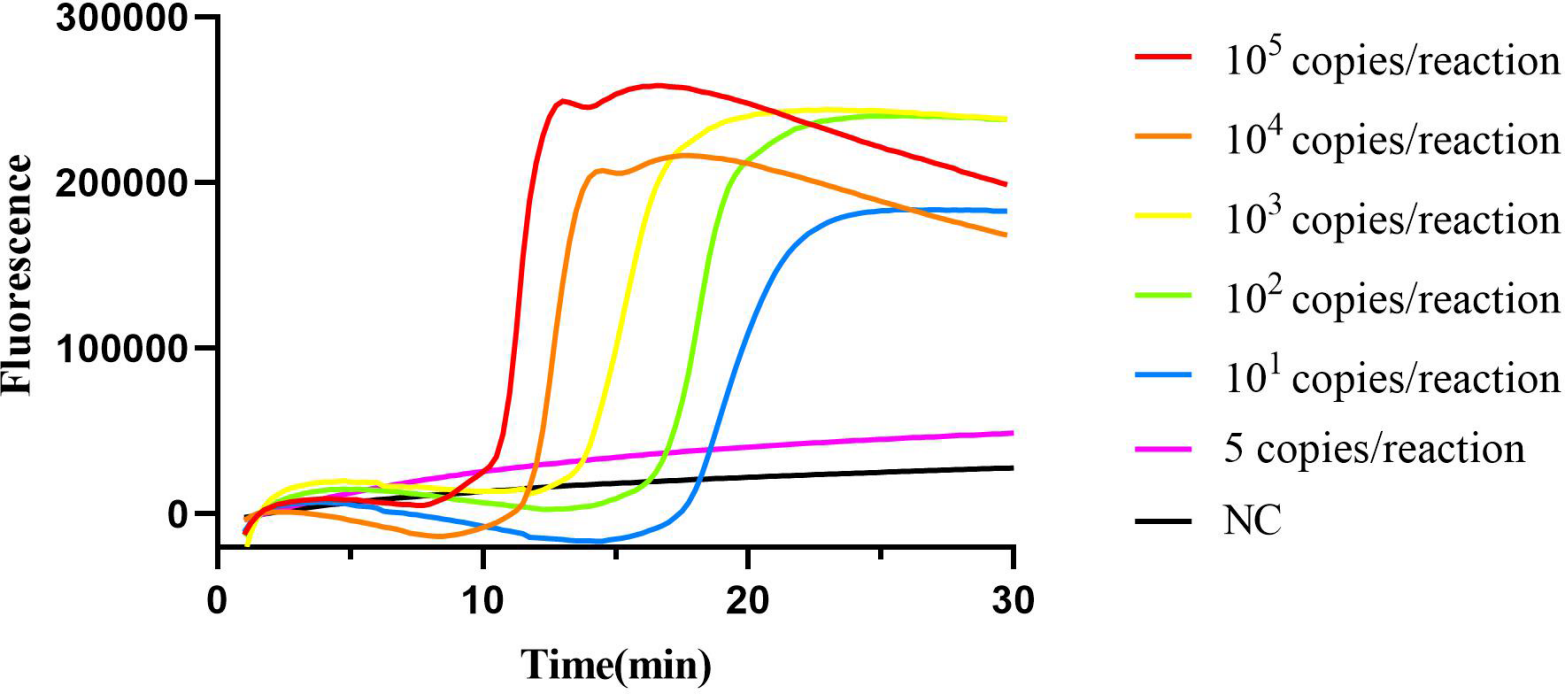
Limit of detection of cyp141-RealAmp assay.

The 10-fold serially diluted standard plasmid of the cyp141 gene ranging from 1×10^5^ copies was subjected to the cyp141-RealAmp reaction system. The lowest concentration of cyp141 that can be detected by the cyp141-RealAmp is 10 copies per tube in 30 min. The real-time fluorescence curves for various concentrations of cyp141 and the blank are displayed in **Figure 3**.

### 3.3 cyp141-RealAmp for evaluation of sputum samples

For detection and evaluation, a collection of 213 clinical sputum samples was obtained from patients. Among the 169 patients (Group A-D), 97 (57.40%, 95% CI: 49.94–64.86) were found to be AFB positive, 111 (65.68%, 95% CI: 58.52–72.84) were culture positive, cyp141-RealAmp was positive in 157 (92.90%, 95% CI: 89.03–96.07), and Xpert MTB/RIF could detect TB in 160 (94.67%, 95% CI: 91.28–98.06). There was no statistical difference between the detection rate of cyp141-RealAmp and that of Xpert MTB/RIF (*P* > 0.05), but both were statistically higher than that of culture (*P*< 0.05) and AFB (*P*< 0.05). A total of 44 control cases (Group E) tested negative for cyp141-RealAmp and Xpert MTB/RIF. In the A+C+, 70 cases tested positive for cyp141-RealAmp (70/71). In A+C– and A-C+, the detection rates of cyp141-RealAmp and Xpert MTB/RIF were the same, both 93.51% and 97.50%, respectively. Xpert MTB/RIF identified 26 out of 32 patients (81.25%, 95% CI 67.73–94.77) who were diagnosed clinically and radiologically as (A-C-), while cyp141-RealAmp detected 24 patients (75.00%, 95% CI 60.00–90.00) (**Table 3**).

**Table 3:** Group-Wise Comparison of Xpert MTB/RIF and cyp141-RealAmp Assay.

Group	Status	Xpert MTB/RIF	Real-Amp	Detection rate (95% CI)	
				Xpert MTB/RIF	cyp141-RealAmp
A	A+C+ (n = 71)	71	70	100 (93.60–100)	98.59 (93.51–100)
B	A+C– (n = 26)	24	24	93.51 (82.07–100)	93.51 (82.07–100)
C	A–C+ (n = 40)	39	39	97.50 (92.66–100)	97.50 (92.66–100)
D	A–C– (n = 32)	26	24	81.25 (67.73–94.77)	75.00 (60.00–90.00)
E	Control (n = 44)	0	0		

CI, confidence interval; A+C+, AFB positive, culture positive; A+C–, AFB positive, culture negative; A–C+, AFB negative, culture positive; A–C–, AFB negative, culture negative; E group, suffering from respiratory diseases other than TB, in whom TB was excluded on the basis of clinical symptom, radiological examination, and microbiological tests, this includes 8 cases of non-tuberculous mycobacterial disease.

### 3.4 Comparative analysis of cyp141-RealAmp and Xpert MTB/RIF

**Table 4** shows that cyp141-RealAmp had a positive predictive value of 100% (95% CI 96.84–100) and a negative predictive value of 78.57% (95% CI 67.82–89.32). Based on *Kappa* statistics, cyp141-RealAmp and Xpert MTB/RIF showed “highly significant” concordance (*Kappa* = 0.89) (**Table 5**).

**Table 4:** Comparison of Xpert MTB/RIF and cyp141-RealAmp.

	Sensitivity (95% CI)	Specificity (95% CI)	PPV (95% CI)	NPV (95% CI)
Xpert MTB/RIF	94.67 (91.28–98.06)	100 (89.78–100)	100 (97.08–100)	83.02 (72.91–93.13)
cyp141-RealAmp	92.90 (89.03–96.07)	100 (89.78–100)	100 (96.84–100)	78.57 (67.82–89.32)

CI, confidence interval; NPV, negative predictive value; PPV, positive predictive value.

**Table 5:** Efficacy and concordance between Xpert MTB/RIF and cyp141-RealAmp for clinical sputum samples.

cyp141-RealAmp	Xpert MTB/RIF		Total	*Kappa*	*P*
	Positive	Negative			
Positive	154	3	157	0.89	>0.05
Negative	6	50	56		
Total	160	53	213		

## 4 Discussion

Tuberculosis has emerged as a significant global health issue (Liebenberg et al., 2022). The treatment duration and anti-tuberculosis medications required vary depending on the infecting strain. Therefore, rapid detection and differentiation between MTB and *M. bovis* are crucial for the prevention and treatment of tuberculosis (Goletti et al., 2022). This research details the development of a LAMP assay deployed on a fluorescence amplification platform with precise temperature control, tailored for MTB diagnosis from sputum specimens. The findings indicated that cyp141-RealAmp reliably discerns the presence of MTB by specifically targeting the 1–1006 bp region of the cyp141 gene, thereby distinguishing it from the genomes of M. bovis or BCG. The present study will contribute to the development of novel clinical diagnostic strategy for the rapid identification of MTB.

The cyp141-RealAmp assay developed in this study effectively overcomes the drawbacks of these traditional methods. Traditional MTB identification techniques include Ziehl-Neelsen staining (Wang et al., 2016), solid culture (Paradkar et al., 2023), tuberculin skin test (Arya et al., 2018), tuberculosis antibody detection (Wu et al., 2014), and liquid culture (Walters et al., 2017), but all have some limitations, such as low sensitivity, low specificity, and long turnaround time (Suarez et al., 2019). Previously, the WHO-approved cartridge-based PCR test, targeting the rpoB gene of MTB, is considered a reliable diagnostic tool for tuberculosis (Park et al., 2013); however, this method is costly and requires specialized equipment and trained personnel, which may limit its use in remote or resource-limited areas. The cyp141-RealAmp eliminates the need for complex and pricey equipment while allowing for a quick diagnosis of TB. More importantly, the cyp141-RealAmp method can specifically identify MTB, a capability lacking in Xpert MTB/RIF (de Vos et al., 2021).

Additionally, the cyp141-RealAmp method developed in this study exhibits high sensitivity (10 copies per reaction). However, Mo’s findings demonstrate even higher sensitivity (6 copies per reaction) (Mo et al., 2017), possibly due to the utilization of more expensive equipment and kit. Nevertheless, considering the balance between sensitivity and cost-effectiveness, our detection method remains competitively advantageous.

Concerning the detection of A+C+ samples, our method exhibited comparable specificity to the Xpert MTB/RIF method. It is worth mentioning that the positivity detection rate of cyp141-RealAmp was 98.59%, while that of Xpert MTB/RIF was 100%; this is because one M. bovis positive sample, detected as negative by cyp141-RealAmp, was detected as positive by Xpert MTB/RIF, further demonstrating the specificity of cyp141-RealAmp for MTB. Previous studies using primers designed from the RV510 gene also achieved 100% specificity in identification (Sales et al., 2015). However, they employed conventional amplification protocols with relatively complex temperature profiles and higher instrument requirements (Sales et al., 2015). In contrast, this study utilized the RealAmp isothermal amplification protocol, which has lower instrument requirements and costs, thus possessing greater potential for clinical application.

Furthermore, the cyp141-RealAmp detection method demonstrates remarkably high accuracy (92.90%). In the detection of A+C– and A–C+ samples, both cyp141-RealAmp and Xpert MTB/RIF exhibit similar detection performance. Our findings were comparable to the detection rate reported in a study targeting the IS6110 gene, where LAMP achieved a detection rate of 98.4% in A+C+ samples (Sethi et al., 2013). In contrast, our study observed a notably detection rate of 97.50%, which significantly surpasses the 76.9% detection rate reported in the a for mentioned study in A–C+ sputum samples (Sethi et al., 2013). For A–C– group samples, the positivity detection rate of cyp141-RealAmp is 75.00%. Importantly, whereas clinical sputum identification of A–C– samples required approximately 8 weeks, cyp141-RealAmp can provide rapid detection results within 30 minutes. Furthermore, our research strongly indicates that even in AFB-negative cases, the LAMP method demonstrates high sensitivity. This contrasts with the findings of Byae et al. who suggested that LAMP is only valuable in AFB-positive samples, as they did not detect any additional true positive cases nor cases undetectable by AFB (Gelaw et al., 2017).

The cyp141-RealAmp assay showed remarkable consistency and efficacy compared to the Xpert MTB/RIF detection method. Although the cyp141-RealAmp assay established in our study shows high performance, its validation was limited to sputum samples. Further validation in other sample types like pleural fluid and feces may be needed. Moreover, preparing the detection reagents in a freeze-dried form could enhance their transportability and suitability for primary healthcare settings.

## 5 Conclusion

In summary, the cyp141-RealAmp test described in this study proves highly advantageous for identifying MTB in sputum samples. It obviates the necessity for intricate and costly equipment, facilitating rapid TB diagnosis. Considering the imperative role of a prompt and accurate diagnostic approach in combating MTB, the straightforward, swift, and cost-effective cyp141-RealAmp assay elucidated in this research holds promise for broadening access to MTB testing and contributing to global TB eradication efforts.

## Data availability statement

The datasets presented in this study can be found in online repositories. The names of the repository/repositories and accession number(s) can be found below: NCBI (https://www.ncbi.nlm.nih.gov/), AL123456. And the data that support the findings of this study are available from the corresponding author, HZ, upon reasonable request.

## Ethics statement

The studies involving humans were approved by This study was approved by the ethics committee of This study was approved by the ethics committee of Nanjing Center for Disease Control and Prevention (reference number: PJ2020-A001-04). The studies were conducted in accordance with the local legislation and institutional requirements. The human samples used in this study were acquired from a by- product of routine care or industry. Written informed consent for participation was not required from the participants or the participants’ legal guardians/next of kin in accordance with the national legislation and institutional requirements.

## Author contributions

YZ: Writing – original draft. ZF: Writing – original draft. YX: Writing – review & editing, Conceptualization, Validation, Resources. SL: Writing – original draft. RZ: Writing – original draft. XS: Writing – review & editing. XW: Writing – original draft. HZ: Writing – review & editing.
