# Supplementary material for: Rapid detection of Mycobacterium tuberculosis based on cyp141 via real-time fluorescence loop-mediated isothermal amplification (cyp141-RealAmp)
Source: Front Cell Infect Microbiol. 2024 Jun 13;14:1349063. doi: 10.3389/fcimb.2024.1349063 (PMC11208306; doi:10.3389/fcimb.2024.1349063)
Supplement: Supplementary file 1 [file Table_1.docx]

**Table S1** the basic information of all study participant

|  |  | TB group  (n=169) | Control group  (n=44) | Sampling location |
| --- | --- | --- | --- | --- |
| Sex | Male(n=152) | 122(72.19%) | 30(68.18%) | The Second Hospital of Nanjing |
|  | Female(n=61) | 47(27.81%) | 14(31.81%) |  |
| Average age |  | 55.92±19.02 | 58.81±18.27 |  |
